# Genetic targeting of the endoderm with claudin-6^CreER^

**DOI:** 10.1002/dvdy.21437

**Published:** 2008-01-16

**Authors:** William J Anderson, Qiao Zhou, Victor Alcalde, Osamu F Kaneko, Leah J Blank, Richard I Sherwood, J Sawalla Guseh, Jayaraj Rajagopal, Douglas A Melton

**Affiliations:** Department of Molecular and Cellular Biology, Howard Hughes Medical Institute, Harvard Stem Cell Institute, Harvard UniversityCambridge, Massachusetts

**Keywords:** endoderm, Cldn6, Cre-ER, embryonic stem cells, β cells, organogenesis, gene targeting, lineage analysis

## Abstract

A full description of the ontogeny of the β cell would guide efforts to generate β cells from embryonic stem cells (ESCs). The first step requires an understanding of definitive endoderm: the genes and signals responsible for its specification, proliferation, and patterning. This report describes a global marker of definitive endoderm, Claudin-6 (Cldn6). We report its expression in early development with particular attention to definitive endoderm derivatives. To create a genetic system to drive gene expression throughout the definitive endoderm with both spatial and temporal control, we target the endogenous locus with an inducible Cre recombinase (Cre-ER^T2^) cassette. *Cldn6* null mice are viable and fertile with no obvious phenotypic abnormalities. We also report a lineage analysis of the fate of *Cldn6*-expressing embryonic cells, which is relevant to the development of the pancreas, lung, and liver.

## INTRODUCTION

During gastrulation, pluripotent cells of the epiblast become specified to form one of the three embryonic germ layers: ectoderm, mesoderm, or definitive endoderm. The definitive endoderm gives rise to the epithelia of numerous organs including the thyroid, lung, liver, stomach, pancreas, and intestines. In the past decade, the potential use of embryonic stem cells (ESCs) as cell based therapeutic vehicles to treat diseases has been amply discussed. Current investigations are underway in numerous laboratories to elucidate paracrine factors that can direct differentiation of ESCs to definitive endoderm, which can then be further instructed to differentiate into an organ progenitor cell, and ultimately to a fully differentiated cell type of interest (such as hepatocytes of the liver, epithelial cells of the lung, or β cells of the pancreas).

This presents the challenge of understanding how definitive endoderm is formed and patterned. Specification of definitive endoderm is a Nodal-dependent process (Schier, 2003) but little is known about endoderm progenitor cells; the cues responsible for their expansion and the signals that pattern the gut tube along the anterior-posterior axis are yet unknown. Tools that would enable the genetic manipulation of the definitive endoderm will help to better understand these processes. To that end, we have sought a marker that would target definitive endoderm during development. A promising candidate arose from an in situ hybridization screen performed by Rosa Beddington's group (Sousa-Nunes et al., 2003). They examined expression of genes from an E7.5 endoderm cDNA library, which was derived from visceral and definitive endoderm, and the node-tissues responsible for specification of the axial mesendoderm. One clone, r8707a53, was expressed in the forebrain, visceral yolk sac, otic vesicle, and throughout the entire definitive endoderm at E9.0. This clone encoded a member of the Claudin superfamily of proteins, Claudin-6.

Claudins are the major structural proteins involved in tight junction formation. Currently in the mouse, there are 24 genes in the Claudin superfamily. Based on expression patterns, it is possible that Claudins come in two varieties: “housekeeping” Claudins and tissue-specific Claudins (Van Itallie and Anderson, 2006).

*Cldn6* was first identified by searching for sequences in genomic databases that were similar to *Cldn1* and *Cldn2*. It was isolated by PCR from mouse kidney and found to encode a protein of 219 amino acids in length with a molecular mass of 23.4 kDa (Morita et al., 1999). Cldn6 has been subsequently studied both in vitro and in vivo (Turksen and Troy, 2001; Turksen and Troy, 2002; Troy et al., 2005; Arabzadeh et al., 2006). While its functional role in the epidermal permeability barrier is of importance, our interest in Cldn6 is as a marker of endoderm and a means to manipulate gene expression therein.

## RESULTS AND DISCUSSION

### Expression of Claudin-6, a Global Marker of Definitive Endoderm

**Fig. 1:**
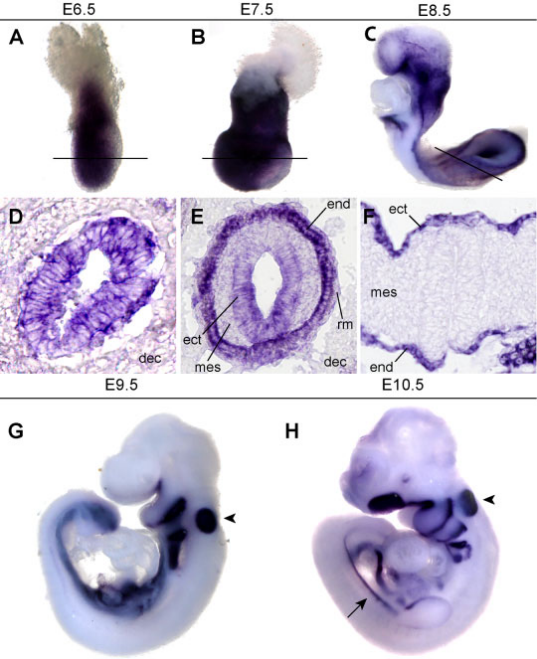
Expression of *Cldn6* from commencement of gastrulation to the early stages of gut tube organogenesis. **A,D:** Expression at E6.5 is quite broad throughout the epiblast, with the exception of the primitive streak and visceral endoderm. Similar expression is seen at E7.5 (**B,E**). **C,F:** By E8.5, expression begins to be restricted to the endoderm and by E9.5 (**G**) and E10.5 (**H**), expression is restricted to the endoderm, otic vesicle (arrowhead), and mesonephros (arrow). A–C are representative whole mount stainings for the sections in D–F), respectively. Embryos in D–F were sectioned in their deciduas. Plane of section is indicated by a line in the corresponding whole mount embryo. For E, posterior is facing up. dec, decidua; ect, ectoderm; mes, mesoderm; end, endoderm; rm, Reichert's membrane; nf, neural fold; he, heart.

We examined expression of *Cldn6* by in situ hybridization, in sections and whole mounts, from gastrulation to the initial stages of organogenesis (Fig. 1). At E6.5, *Cldn6* is broadly expressed throughout the epiblast as well as the hypoblast. It is absent in the primitive streak (Fig. 1A) and in some visceral endoderm surrounding the epiblast (Fig. 1A,D). This lack of expression in the primitive streak is not surprising, as cells undergo an epithelial-to-mesenchymal transition (EMT) as they migrate to form mesoderm and definitive endoderm, thus dissolving adherens and tight junctions (Solursh and Revel, 1978; Tam et al., 1993; Cano et al., 2000; Batlle et al., 2000; Zohn et al., 2006; Ikenouchi et al., 2003; Ohkubo and Ozawa, 2004). At E7.5, expression is still evident throughout most of the epiblast (Fig. 1B,E). The primitive streak and nascent mesoderm are devoid of *Cldn6* expression (Fig. 1E). By E8.5, expression begins to become restricted to the definitive endoderm (Fig. 1C,F). This is the stage when initial patterning of the gut tube along the anterior-posterior axis is thought to occur. At E9.5, intense expression is seen in the entire gut, otic vesicles, and a small region of the forebrain (Fig. 1G). This expression pattern at E9.5 is comparable to what is reported by others (Gitton et al., 2002; Sousa-Nunes et al., 2003). At E10.5, expression is similar to E9.5, but staining in the mesonephros and forebrain is now more apparent (Fig. 1H).

**Fig. 2:**
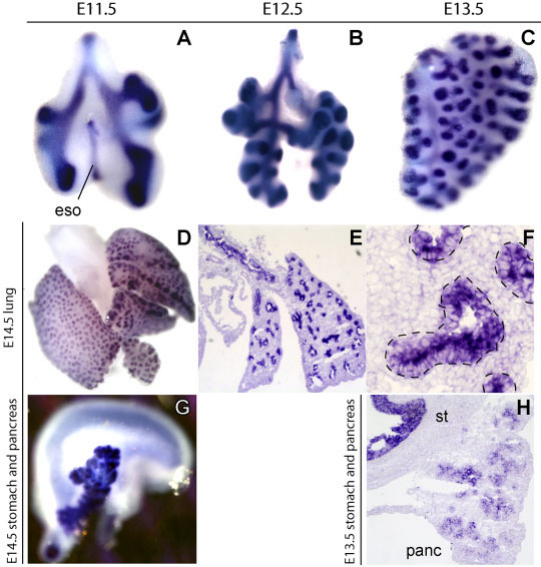
Epithelial-restricted expression of *Cldn6* in the developing lung and pancreas. **A–D:** Whole mount in situ hybridization shows *Cldn6* expression throughout the entire epithelia of the lung from E11.5 to E14.5 [(A) E11.5, (B) E12.5, (C) E13.5, (D) E14.5)]. **E,F:** Expression of *Cldn6* in the E14.5 lung is restricted to the epithelia with no mesenchymal expression. E: Expression of *Cldn6* can be seen in the tracheal epithelia, as well as both the proximal and distal airways. F: A higher magnification view of staining from a different lung. Epithelia is outlined by a black dashed line. **G:** Expression of *Cldn6* in the E14.5 pancreas. As with the lung, expression is restricted to the epithelia. Staining in the epithelial lining of the stomach can be seen faintly. **H:** *Cldn6* expression in the E13.5 pancreas. The pancreas is still attached to the stomach. Strong epithelial staining can be seen in the branching epithelia. panc, pancreas; st, stomach.

To examine whether *Cldn6* maintains epithelial expression in endoderm-derived organs, we performed whole mount and section in situ hybridization on embryonic lung and pancreas. *Cldn6* is strongly expressed in the epithelia of both organs (Fig. 2A–F, 2G–H, pancreas). On sections, no staining in the mesenchyme is evident (Fig. 2E,F,H).

To summarize, *Cldn6* is expressed broadly at early stages of development. However, by E9.5, expression of *Cldn6* is largely restricted to definitive endoderm along its entire length, making it a good candidate as a global endoderm marker. Other markers that have been used to mark endoderm include *Shh, FoxA* genes, and *Sox17. Shh*, while initially expressed throughout the definitive endoderm, is downregulated in the prospective pancreatic region (Apelqvist et al., 1997). Of the *FoxA* genes, *FoxA3* is not expressed in the most anterior endoderm derivatives, while *FoxA1* and *FoxA2* are expressed throughout the definitive endoderm as well as other tissues such as the notochord and neural tube (Monaghan et al., 1993). *Sox17*, while expressed in nascent endoderm, is downregulated in the endoderm at stages post E7.5 and is expressed in other tissues such as vasculature (Kanai-Azuma et al., 2002; Matsui et al., 2006; unpublished observations). This makes *Cldn6* one of the few genes that allows for targeting of the definitive endoderm throughout the entire anterior-posterior axis before and after gut tube patterning (E8.0 onward).

### Expression of *Cldn6* in the Adult Mouse

We investigated whether *Cldn6* was expressed in epithelial structures in the adult. Adult mice of both sexes were sacrificed and organs were harvested and transcriptional analysis was performed by RT-PCR. Out of the 23 tissues examined, *Cldn6* was present in 11 tissues (see Supplemental Fig. 1, which can be viewed at www.interscience.wiley.com/jpages/1058-8388/suppmat). Using *β-actin* as a loading control, we can make a semi-quantitative assessment of relative expression levels. *Cldn6* is most highly expressed in the olfactory epithelium, followed by the coagulating gland, kidney, eye, trachea, prostate, epididymis, lung, liver, eye gland, and uterine horns. Immunohistochemistry with goat anti-Cldn6 polyclonal antibodies, however, failed to detect Cldn6 in lung, trachea, kidney, or testis (data not shown). These results are in broad agreement with previous reports that *Cldn6* expression in the adult is very weak or absent (Morita et al., 1999; Turksen and Troy, 2004).

### Generation of the *Cldn6*^*CIHV*^ Allele

Based on its expression during early development, we hypothesized that the *Cldn6* gene would be useful to drive expression throughout the entire endoderm. One genetic method that has been used extensively for gain of function or loss of function studies is the Cre-ER system (Branda and Dymecki, 2004). Applications of this system can include activation or removal of target genes in a spatially and temporally controlled manner.

A multifunctional cassette was generated consisting of the *Cre-ER*^*T2*^ gene for genetic manipulation, followed by an internal ribosome entry site (IRES), the gene encoding a nuclear localized yellow fluorescent protein variant (the *histone 2B/Venus* fusion) as a reporter for gene expression, a strong transcriptional stop sequence using the SV40 polyadenylation signal to prevent transcriptional read-through, and a floxed selection cassette that self-excises in the male germline. We termed this cassette “CIHV” for *Cre-ER*^*T2*^-IRES-*H2B/Venus* (Zhou et al., 2007).

We first tested this construct by transfection into COS cells. A plasmid was made with the CIHV cassette cloned downstream from a CAGGS promoter. As shown in Supplemental Figure 2, all transfected cells express a nuclear localized YFP. To test whether Cre recombinase activity is tamoxifen-dependent, we co-transfected a plasmid with a CAGGS promoter upstream of a floxed transcriptional stop and cytoplasmic *RFP*. RFP expression was dependent upon addition of 4-hydroxytamoxifen to the culture media, verifying that the Cre-ER^T2^ system is tightly regulated (see Suppl. Fig. 2).

**Fig. 3:**
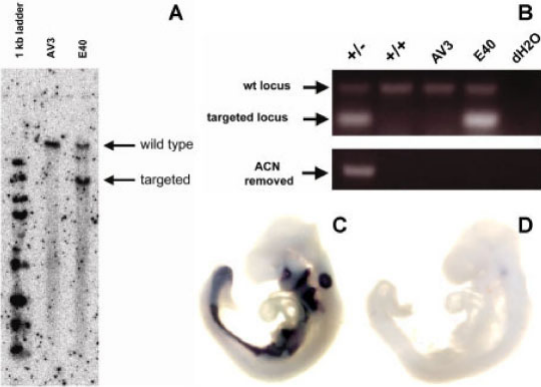
Successful targeting of the *Cldn6* locus with the CIHV cassette. **A:** Southern blot analysis. Upon successful recombination, the entire coding sequence for *Cldn6* is removed, generating a null allele. After NcoI digest, the wild type locus generates a band of approximately 12.5 kb, while the targeted allele has a smaller band close to 8 kb. For reference, the first two bands on the 1-kb ladder are 10 and 8 kb, respectively. As seen in the parental ES cell lane (AV3), only one band is present higher than 10 kb, while in the targeted ES cell clone lane (E40), there is a band at the same height as in the wild type lane, but a second band present at 8 kb. **B:** Genotyping by PCR confirms germline transmission of the CIHV allele. AV3 and E40 refer to the parental ES cell line and targeted ES cell line used for blastocyst injection, respectively. The upper gel distinguishes between the wild type and targeted locus. The lower gel is a PCR to demonstrate *ACN* cassette removal during germline transmission. **C,D:** *Cldn6*^*CIHV/CIHV*^ mice are null mutants for *Cldn6*. In situ hybridization for *Cldn6* in embryos from a *Cldn6*^*CIHV/+*^ intercross. C: *Cldn6*^*CIHV/+*^; D: *Cldn6*^*CIHV/CIHV*^. As shown in D, *Cldn6* mRNA is absent in the *Cldn6*^*CIHV/CIHV*^ embryo.

AV3 mouse ES cells (a gift from Jill McMahon) were targeted with the CIHV cassette (Suppl. Fig. 3). Out of a total of 1,474 colonies screened, one clone (E40) successfully underwent homologous recombination at the *Cldn6* locus (Fig. 3A,B). Chimeras were generated by blastocyst injection and transmitted the allele through the germline (Fig. 3B). Heterozygotes from this cross were renamed *Cldn6*^*CIHV/+*^.

### A Genetic Lineage Analysis of Cldn6^+^ Cells at Different Time Points in Development

To test Cre activity, we crossed *Cldn6*^*CIHV/+*^ males to homozygous ROSA26 Reporter (R26R) females (Suppl. Fig. 4A). The R26R line has a floxed transcriptional stop sequence followed by the neomycin resistance/lacZ fusion gene β*geo* inserted into the *ROSA26* locus, a non-coding open reading frame in the mouse that is ubiquitously expressed in all tissues (Soriano, 1999). This cross permits indelible labeling of cells that express *Cldn6* at the time of tamoxifen injection and all of their progeny.

**Fig. 4:**
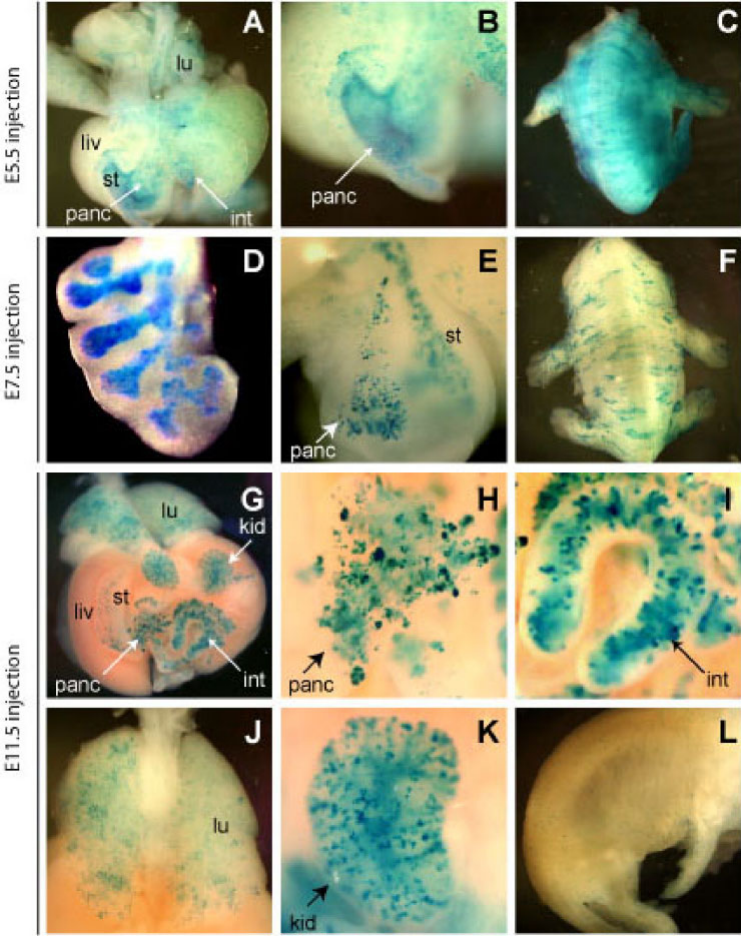
Lineage analysis of *Cldn6*^*+*^ cells at different times in embryonic development. Tamoxifen injection at E5.5 shows widespread labeling throughout the embryo (**A–C**). Tamoxifen injection at E7.5 and later shows epithelial-restricted expression of β-galactosidase in definitive endoderm derivatives (**D,E, G–J**). Epithelia of the kidney is also labeled (G, **K**). Melanocytes also label (C, **F**, scattered cells in **L**), with a decrease in labeling correlating to later times of tamoxifen injection. Embryos administered tamoxifen at E5.5 (A–C) and E7.5 (D–F) were harvested at E12.5 for analysis. Embryos administered tamoxifen at E11.5 (G–L) were harvested at E15.5 for analysis. panc, pancreas; lu, lung; liv, liver; st, stomach; kid, kidney; int, intestine.

We experimented with tamoxifen dosage as was done previously (Hayashi and McMahon, 2002) and found that a 4-mg injection of tamoxifen to pregnant females provided maximum labeling of embryos without significant embryonic loss (between 0–2 embryos resorbed per litter). We then performed a time course of tamoxifen injection during embryonic development, which is schematized in Supplemental Figure 4B. Four milligram tamoxifen injections were administered to pregnant females between E5.5 and E12.5, with embryos whose mothers were injected between E5.5 and E10.5, harvested on E12.5, and those whose mothers were injected on E11.5 and E12.5, harvested on E15.5. Selected whole mount images are shown in Figure 4. At all stages examined, neither Cre-independent β-galactosidase expression nor tamoxifen-independent β-galactosidase expression was detected (data not shown). Embryos at each stage of injection were sectioned post-staining to better visualize localization of the signal to the epithelial or mesenchymal compartments (data not shown).

**Fig. 5:**
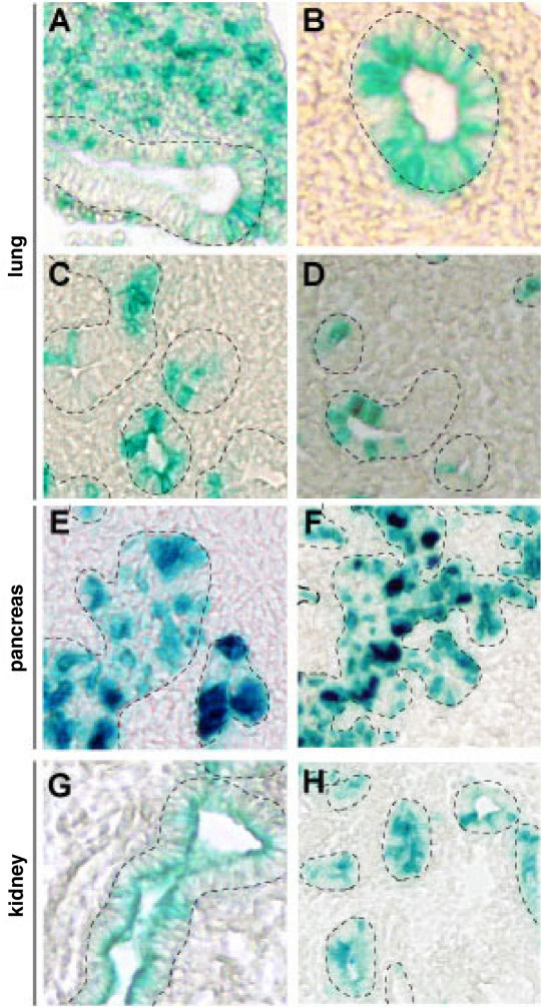
Injection of tamoxifen post-E7.5 results in epithelial-specific expression of β-galactosidase. In all images, epithelia is outlined in black dashed lines. **A–D:** In the E15.5 lung, injection at E5.5 (A) or E6.5 (B) labels mesenchymal cells, yet injections at E7.5 and later, shown here at E11.5 (C) or E12.5 (D), only label epithelia. Stage of harvesting: (A,B), E12.5; (C,D), E15.5. **E,F:** In the E12.5 pancreas, injection at E6.5 (E) leads to strong staining in the epithelia as well as in some scattered mesenchyme. Injection at E7.5 (F) is restricted to the epithelia. No mesenchymal staining was seen in any section examined. **G,H:** In the E15.5 kidney, injection at E11.5 (G) and E12.5 (H) are shown. All magnification at 10× except F at 20×. inj., injection.

Tamoxifen injection to pregnant females at E5.5 labels cells throughout the embryo, both in the epithelia and mesenchyme (Figs. 4A–C, 5A). This is consistent with the ubiquitous expression of *Cldn6* at the time Cre recombinase would be most active (roughly E6.0–7.0). Based on the composite data, it appears E7.5 is the best time point for tamoxifen administration (Fig. 4D–F) if one aims to mostly label definitive endoderm and its derivatives without ubiquitous labeling of the embryo. From this stage forward, Cre-mediated recombination is detected in epithelia including all endoderm-derived organs (Figs. 4D,E,G–J, 5C,F), the kidney (Figs. 4G,K, 5G,H), olfactory epithelia, lens epithelia, and semicircular canal (data not shown). Cells that appear to be melanocytes also label (Fig. 4C,F,L), although *Cldn6* expression in these cells is too low to visualize by in situ hybridization. Based on the kinetics of tamoxifen, E7.5 would be the optimal stage for misexpression in definitive endoderm as Cre recombinase would be active around the time the endoderm is patterned and before organogenesis begins. Of note, given that the degree of Cre-mediated recombination is dependent upon the dose of tamoxifen administered, not all *Cldn6*-expressing cells undergo recombination at the dose used here.

In summary, *Cldn6* is expressed in various epithelia in the developing embryo. It is a more useful definitive endoderm marker than others that have been described to date including the widely used marker Sox17. Using a tamoxifen-inducible form of Cre recombinase, we are able to target the entire endoderm using the *Cldn6*^*CIHV*^ line. Based on the timing of tamoxifen administration, one can target various epithelia in the developing embryo for gain of function or loss of function studies. To our knowledge, this is the first and only Cre-ER line available to globally target the endoderm at stages prior to patterning and during organogenesis without affecting most other tissues.

The *Cldn6*^*CIHV*^ line has distinct advantages compared to other genetically modified mice. While *Shh*^*Cre-ER*^ mice (Harfe et al., 2004) can target definitive endoderm, they cannot target pancreatic endoderm during initial stages of organogenesis. As mentioned earlier, *FoxA3*^*Cre*^ transgenic mice do not target the most anterior definitive endoderm (Monaghan et al., 1993; Lee et al., 2005). A *FoxA2*^*rtTA*^ line was recently reported (Frank et al., 2007). This will prove to be quite useful for crosses with tetracycline operator responder lines. Of note, the authors preserved the *FoxA2* coding sequence, as *FoxA2* is haploinsufficient (Ang and Rossant, 1994; Weinstein et al., 1994). Crossing to Cre responder lines, however, will be somewhat laborious as it involves generation of a triple transgenic. *Sox17*^*Cre-ER*^ mice have not been reported, though they would not be useful for endoderm targeting post-E7.5 due to predominant vascular expression (Matsui et al., 2006; unpublished observations).

### *Cldn6*^*CIHV/CIHV*^ Mice Are Viable and Exhibit No Overt Phenotype

To examine whether Cldn6 plays a role in gastrulation or gut tube morphogenesis, mice heterozygous for the *Cldn6* allele (*Cldn6*^*CIHV/+*^) were mated. Female mice were allowed to give birth and progeny were genotyped by tail biopsy upon weaning. Homozygous null mice (*Cldn6*^*CIHV/CIHV*^) were retrieved in the expected Mendelian ratio by PCR genotyping. These mice show no obvious anatomical phenotypic abnormalities and responded to sound (data not shown).

To ensure that targeting the *Cldn6* locus with the CIHV cassette generated a complete null allele, mice heterozygous for *Cldn6* were again mated and embryos were harvested at E9.5 for in situ hybridization. As seen in Figure 3D, no *Cldn6* mRNA was detected in *Cldn6*^*CIHV/CIHV*^ embryos.

To investigate whether *Cldn6* null mice are able to mate normally, *Cldn6*^*CIHV/CIHV*^ mice were mated and allowed to give birth. Females gave birth to litters of normal size. Newborns looked healthy with no grossly abnormal phenotype (data not shown). Mothers showed no rejection of the pups. Mothers nursed normally and successfully exhibited pup retrieval behavior (data not shown). This would suggest that pheromone detection and olfactory cues, at least with regards to mating and nursing behaviors, are normal in these mice.

Concurrently, E8.5 embryos were dissociated and embryonic germ layers were analyzed by microarray analysis (Sherwood et al., 2007). Upon examining the list for members of the *Claudin* gene family, there are eight different Claudins expressed in the endoderm, with *Cldn6* being the highest relative value (Table 1). The other seven, in order of relative expression, are *Cldn7, Cldn12, Cldn8, Cldn4, Cldn10, Cldn3*, and *Cldn11*. While this type of analysis does not provide cellular resolution, it suggests that other Claudin family members may compensate for loss of Cldn6 in the endoderm. Further analysis by in situ hybridization and double loss of function studies may shed insight onto this mechanism. In addition, *Cldn6*^*CIHV/CIHV*^ mice may have subtle phenotypic abnormalities that will be more difficult to reveal or that may appear more noticeable with age.

**Table 1:** Microarray Analysis of Claudin Gene Expression^a^

Gene	Accession	ES cells	Endoderm	Ectoderm	Mesoderm	EE endoderm	EE mesoderm
CLDN01	1437932_a_at	14	24	38	87	37	15
CLDN02	1417231_at	14	19	15	36	1481	126
CLDN03	1460569_x_at	22	41	16	13	29	17
CLDN04	1418283_at	12	70	8	6	12	7
CLDN05	1417839_at	16	12	15	51	28	111
CLDN06	1417845_at	1504	4606	598	58	6174	72
CLDN07	1448893_at	947	1951	118	23	3762	31
CLDN08	1449091_at	9	198	12	11	17	8
CLDN09	1450524_at	13	15	11	11	12	11
CLDN10	1426147_at	14	51	29	26	16	12
CLDN11	1416003_at	7	38	15	47	12	16
CLDN12	1433781_a_at	803	1564	1726	1634	1504	723
CLDN13	1422920_at	20	16	17	16	17	61
CLDN14	1420345_at	15	13	12	14	15	14
CLDN15	1418920_at	17	15	17	18	17	20
CLDN16	1420434_at	10	10	10	9	11	10
CLDN18	1449428_at	9	9	10	9	11	9
CLDN19	1425727_at	9	8	8	7	8	8
CLDN22	1430237_at	23	19	18	18	22	24
CLDN23	1424409_at	8	12	8	8	9	7

a Expression of different members of the *Claudin* family at E8.5. E8.5 embryos were dissociated and separated by embryonic lineage. cDNA was isolated for each sample and hybridized to Affymetrix microarrays. Numbers reflect relative expression levels. Only members of the *Claudin* family of genes are shown here. In the definitive endoderm, *Cldn6* has the highest expression, followed by *Cldn 7, 12, 8, 4, 10, 3,* and *11.* Accession, accession number for gene in public NCBI database; endoderm, definitive endoderm; EE endoderm, extraembryonic endoderm; EE mesoderm, extraembryonic mesoderm.

### Role of Cldn6 in Gastrulation and Endoderm Development

*Cldn6* null mice appear phenotypically normal based on anatomical criteria and overt behavior. From microarray data, this is most likely due to compensation by another Claudin family member, with Cldn7 as a likely candidate. This does not rule out a possible role for Cldn6 in endoderm development. Functional redundancy has been shown in processes important for gastrulation before. For example, in the frog, Cer1 and Lefty1 both play important roles in blocking BMP and Nodal signaling. In mouse, *Cer1*^*−/−*^ mutants (Simpson et al., 1999; Shawlot et al., 2000; Stanley et al., 2000; Belo et al., 2000) and *Lefty1*^*−/−*^ (Meno et al., 1998) mutants have no overt phenotype at gastrulation. However, *Cer1*^*−/−*^; *Lefty1*^*−/−*^ double mutants develop multiple primitive streaks due to Nodal expression expanding outside of the posterior region of the embryo (Perea-Gomez et al., 2002). Therefore, a *Cldn6*^*−/−*^; *Cldn7*^*−/−*^ mutant might show a severe phenotype, and manifest at gastrulation or perhaps even earlier upon egg cylinder formation.

An application of this genetic system is to target the endoderm for misexpression studies. One means is to use a gain-of-function approach, crossing the *Cldn6*^*CIHV*^ line with different Cre responder lines that constitutively activate different signaling pathways upon removal of a floxed stop cassette. The role of the Notch, Hedgehog, and Wnt signaling pathways in Cldn6-positive cells would be interesting to examine given that many responder lines currently exist (Murtaugh et al., 2003; Jeong et al., 2004; Harada et al., 1999). Further analysis will be necessary to determine what role these different signaling pathways play in the definitive endoderm with regards to both organ specification and regulation of endoderm progenitor cells. The *Cldn6*^*CIHV*^ line, combined with the recently reported *FoxA2*^*rtTA*^ line (Frank et al., 2007), will prove to be useful reagents in this regard. This understanding will lead to more efficient strategies to generate and expand definitive endoderm from ESCs, as well as subsequent differentiation to various organ progenitor cells.

## EXPERIMENTAL PROCEDURES

### In Situ Hybridization

In situ hybridization was performed essentially as described using digoxigenin-labeled probes (Wilkinson, 1992). Tammy-Claire Troy (Ottawa Health Research Institute) kindly provided the Cldn6 in situ plasmid.

### RT-PCR

Organs were harvested from adult female and male ICR mice (Taconic) and stored in RNAlater (Ambion) until use. Total RNA was extracted with a RNeasy Kit (Qiagen).

### Generation of the CIHV Cassette

We generated a construct consisting of *Cre-ER*^*T2*^, followed by an IRES element, *H2B-Venus*, a strong transcriptional stop, and a self-excising drug selection cassette (*ACN*) (construct referred to as CIHV). Pfx Taq polymerase was used to generate 5′ and 3′ homology arms for *Cldn6*. PCR conditions were as follows: 1 μl AV3 (strain 129Sv) genomic DNA, 10 μl 10× PCR buffer, 5 μl Pfx enhancer, 1.5 μl 10 mM dNTPs, 22 μl 3M betaine, 1 μl 50 mM MgSO_4_, 0.65 μl DMSO, 1.5 μl each of 10 μM forward and reverse primers, 5.35 μl dH_2_O, and 0.5 μl Platinum Pfx polymerase (Invitrogen). Thermocycler conditions were as follows: initial denaturation at 94°C for 2 min, followed by 35 cycles of denaturation at 94°C for 10 sec, annealing at 55°C for 30 sec, and extension at 68°C for 2 min plus 10 sec per cycle, with a final extension at 68°C for 7 min. Fragments were TOPO cloned into the pCRII vector (Invitrogen) and confirmed by DNA sequencing (MCB Sequencing Facility). Both arms were subsequently ligated with the CIHV cassette.

### Targeting the Cldn6 Locus With the CIHV Cassette

C6-CIHV vector was linearized and phenol:chloroform extracted. AV3 mouse ES cells (7 × 10^6^ cells/ml; a kind gift from Jill McMahon) in EmbryoMax qualified electroporation buffer (Specialty Media) were electroporated with 40 μg of DNA and selected with 300 μg/ml Gentacin (Invitrogen) for 8–9 days. Roughly 1,000 colonies were picked and subjected to Southern blot with a 995-bp 5′ probe (Sambrook et al., 1989). Correctly targeted colonies were identified (E40). The E40 ES cells were used for blastocyst injection (Genome Manipulation Facility). Eighteen chimeras were born, 11 of which were high-grade chimeras. Three (males D1, D4, and E3) transmitted through the germline from crosses with C57Bl/6J females (Jackson Laboratories).

All animal experiments described in this report have been approved by the Harvard University Institutional Animal Care and Use Committee.

### PCR Genotyping

Genomic DNA isolation from tail biopsy was performed following standard protocols (Laird et al., 1991). Offspring were genotyped by PCR using the following strategy. The allele was genotyped using a forward primer located at the end of the 5′ homology arm (gtCldn6F) with two reverse primers, one located in the *Cre* coding sequence (Cre-R) and the other located in the middle of the endogenous *Cldn6* coding sequence (gtCldn6R1). If the locus was undisrupted, gtCldn6F and gtCldn6R1 would amplify a 294-bp product. If the locus was targeted with the CIHV cassette, the product would be slightly less than 200 bp. To ensure self-excision of the selection cassette, a PCR strategy was designed to check for the presence of the *ACN* cassette. Two forward primers, one in the *Venus* coding sequence and the other in the *ACN* cassette itself were combined with a reverse primer, gtCldn6R2, located in the 3′ homology arm. If the *ACN* cassette was still present, ACN-F and gtCldn6R2 would produce ∼200-bp fragment, while if the *ACN* cassette was excised, the Venus-F and gtCldn6R2 would produce ∼600-bp fragment. In all cases, there was successful *ACN* excision through the male germline. Sequences for the primers used are: ACN-F: 5′–ATC GCC TTC TAT CGC CTT CTT GAC GAG TTC–3′; Cre-R: 5′–CGG ACA GAA GCA TTT TCC AGG TAT GCT CAG–3′; gtCldn6F: 5′–CAC TAC ACA GCC CCC TCA AC–3′; gtCldn6R1: 5′–TGA GGA GGG TGA CAA CAC AG–3′; gtCldn6R2: 5′–GAG GTG GAG CTT GGA CTC AG–3′; Venus-F: 5′–ATC ACT CTC GGC ATG GAC GAG CTG TAC AAG–3′.

### Tamoxifen Administration

Tamoxifen (Sigma) was dissolved in corn oil (Sigma) at a concentration of either 10 or 20 mg/ml and passed through a 0.2-μm filter. Injections were administered to pregnant females intraperitoneally.

### X-gal Staining of *Cldn6*^*CIHV/+*^; *ROSA26*^*Reporter/+*^ Embryos

Timed pregnancies were collected, using E0.5 as the date of plug detection. Embryos were dissected out of the decidua, saving extraembryonic membranes. To ensure penetration, heads were removed and the body was eviscerated. The tissues were fixed in 4% paraformaldehyde in PBS for 1 hr at 4°C, followed by two PBS washes for 15 min each. β-galactosidase staining was performed using the HistoMark X-gal Substrate Set (Kirkegaard & Perry Laboratories). Incubations were continued overnight at room temperature for maximal sensitivity.
